# Selection of the Optimal L-asparaginase II Against Acute Lymphoblastic Leukemia: An In Silico Approach

**DOI:** 10.2196/29844

**Published:** 2021-09-08

**Authors:** Adesh Baral, Ritesh Gorkhali, Amit Basnet, Shubham Koirala, Hitesh Kumar Bhattarai

**Affiliations:** 1 Department of Biotechnology Kathmandu University Dhulikhel Nepal

**Keywords:** L-asparaginase II, acute lymphoblastic leukemia, leukemia, cancer, enzyme kinetics, binding affinity, homology modeling, docking, molecular biology, structural biology, protein chemistry, biochemistry

## Abstract

**Background:**

L-asparaginase II (asnB), a periplasmic protein commercially extracted from *E coli* and *Erwinia*, is often used to treat acute lymphoblastic leukemia. L-asparaginase is an enzyme that converts L-asparagine to aspartic acid and ammonia. Cancer cells are dependent on asparagine from other sources for growth, and when these cells are deprived of asparagine by the action of the enzyme, the cancer cells selectively die.

**Objective:**

Questions remain as to whether asnB from *E coli* and *Erwinia* is the best asparaginase as they have many side effects. asnBs with the lowest Michaelis constant (Km; most potent) and lowest immunogenicity are considered the most optimal enzymes. In this paper, we have attempted the development of a method to screen for optimal enzymes that are better than commercially available enzymes.

**Methods:**

In this paper, the asnB sequence of *E coli* was used to search for homologous proteins in different bacterial and archaeal phyla, and a maximum likelihood phylogenetic tree was constructed. The sequences that are most distant from *E coli* and *Erwinia* were considered the best candidates in terms of immunogenicity and were chosen for further processing. The structures of these proteins were built by homology modeling, and asparagine was docked with these proteins to calculate the binding energy.

**Results:**

asnBs from *Streptomyces griseus*, *Streptomyces venezuelae*, and *Streptomyces collinus* were found to have the highest binding energy (–5.3 kcal/mol, –5.2 kcal/mol, and –5.3 kcal/mol, respectively; higher than the *E coli* and *Erwinia* asnBs) and were predicted to have the lowest Kms, as we found that there is an inverse relationship between binding energy and Km. Besides predicting the most optimal asparaginase, this technique can also be used to predict the most optimal enzymes where the substrate is known and the structure of one of the homologs is solved.

**Conclusions:**

We have devised an in silico method to predict the enzyme kinetics from a sequence of an enzyme along with being able to screen for optimal alternative asnBs against acute lymphoblastic leukemia.

## Introduction

Acute lymphoblastic leukemia is a malignant cancer of the white blood cells characterized by uncontrolled overproduction and accumulation of lymphoid progenitor cells [1]. It is most common among children, which compromise 80% of the worldwide acute lymphoblastic leukemia occurrences, although some cases in adults are also seen. It is equally life-threatening in both cases. In the United States, acute lymphoblastic leukemia is estimated to have a frequency of 1.7 cases per 100,000 people [2]. In 2015 alone, 111,000 deaths were reported out of 876,000 cases worldwide [3]. Thus, a substantial potential market exists for new and improved therapies to acute lymphoblastic leukemia.

Experiments in the 1950s with guinea pig serum have shown that it could inhibit the growth of transplantable lymphoblastic tumors in mice and rats along with radiation-induced leukemia in mice [4]. Research linked this effect to guinea pig serum being rich in L-asparaginase [5], a nonhuman enzyme of often bacterial origin, belonging to the amidase group that hydrolyses the amide bond in L-asparagine to form L-aspartic acid and ammonia [6]. It has since been shown to be an effective antineoplastic agent and is often used in conjugation with chemotherapy for acute lymphoblastic leukemia treatment.

Normal cells require L-asparagine as an amino acid for the synthesis of proteins. A natural diet like vegetables is one of the sources of L-asparagine for the body. It is not classified as an essential amino acid as it is naturally synthesized by the body through a pathway involving the enzyme L-asparagine synthase, which coverts aspartic acid and glutamic acid into L-asparagine [7]. Neoplastic cells like acute lymphoblastic leukemia cells lack this enzyme and therefore are not able to produce L-asparagine on their own [8]. This leaves them dependent on L-asparagine from outside sources like the serum where it is pooled from diet and from normal cells. This provides the basis for the use of L-asparaginase as a therapeutic agent against acute lymphoblastic leukemia, the intent being to deplete the local circulating pools of L-asparagine in the blood serum thus starving the cancer cells of the amino acid and causing cell death.

L-asparaginase is produced by a wide variety of organisms and can be classified into several families. The ones of therapeutic interest can consist of two enzymes called L-asparaginase of two closely related families named L-asparaginase I and L-asparaginase II. L-asparaginase I, referred to also as asnA, is a low-affinity enzyme found in the cytoplasm and is constitutively produced by the organism. L-asparaginase II, referred to as asnB, on the other hand, is a high-affinity periplasmic enzyme expressed during anaerobiosis. Its expression is dependent on aeration, carbon source, and amino acid availability [9].

Extracellular L-asparaginase accumulates in the culture broth and thus is most favorable for extraction and downstream processing for commercial production [10]. The most commercial form of therapeutic L-asparaginase is extracted from *E coli* and *Erwinia* species. They secrete the enzyme into the periplasmic space between the plasma membrane and the cell envelope [11]. The enzyme is extracted by lysis of the cells, which brings the enzyme along with inner cell contents into the culture medium. It is usually purified using fractionation with ammonia sulfate.

However, the commercially available L-asparaginase has several drawbacks. L-asparaginase from *E coli* and *Erwinia* is known to show immunogenic and allergic reactions. Most therapeutic use of L-asparaginase has shown toxicity [12]. Toxicity of L-asparaginase can be attributed to lower activity of the enzyme to L-asparagine and higher activity to glutamine. Thus, the decrease in glutamine levels in the normal cells causes an allergic reaction [13]. Another problem with the currently available L-asparaginase is the immunological response. The body recognizes the enzyme as being foreign and thus mounts an immune response against the enzyme, which can range from a mild allergic reaction to anaphylactic shock [14].

The Michaelis constant (Km) is a value for the substrate concentration at which the reaction rate is half of the maximum reaction rate. A lower Km suggests that the enzyme can reach half the maximum reaction rate at lower substrate concentrations. One can interpret this to mean that enzymes with lower Km have greater activity toward that substrate. An enzyme with greater activity toward L-asparagine can be expected to show fewer undesirable effects, as it will have a lower activity to unintended substrates [15]. Another useful metric for the measurement of enzyme activity is k_cat_ or the turnover number. It gives the number of substrates converted to a product by a single molecule of enzyme per unit time. The turnover number signifies the rate at which a substrate is catalyzed by the enzyme [16].

Catalysis is based on binding energy that lowers the activation energy and overcomes the unfavorable entropic requirements needed for the correct orientation of the catalyst and reactants brought together for reaction [17]. Binding energy is the energy released when a substrate forms weak bonds with the enzyme active site. Binding energy is measured as the free energy (Delta G). Gibbs free energy, defined as “a thermodynamic potential that measures the capacity of a thermodynamic system to do maximum or reversible work at a constant temperature and pressure (isothermal, isobaric), is one of the most important thermodynamic quantities for the characterization of the driving forces” [18].

Experimental calculation of this energy is difficult and cumbersome. Thus, experimental screening techniques for a lead compound for drug candidates are still expensive and slow despite several advances in automation and parallelization of the process. A more efficient method would be to screen a large library of small molecules in silico before short-listing a small group for experimental verification. The availability of large volumes of experimental data on the 3D structure of the enzymes and their substrates allows us to analyze their interaction. Docking is one of these in silico methods where rigid body interaction of contact surfaces of the ligand or small molecules and the target protein is determined using computational methods. Combinatorial methods are used to account for the ligand conformational flexibility, and various energy functions are used to calculate energetics of the interaction. Docking is typically used to screen for potential lead compound candidates from a large library of small molecules based on their binding energy and other parameters to the target protein. Those compounds with greater binding energy to the protein are seen as potential inhibitors and thus considered to lead for developing drugs of therapeutic value [19]. However, in L-asparaginase–based therapy of acute lymphoblastic leukemia, the enzyme itself is used as a therapeutic agent, while the substrate, L-asparagine, is the target compound. Our goal in this research is to find a better enzyme candidate with more favorable interaction with our target compound. Thus, our use of docking in this research is different from the standard use of the docking method. We used docking to screen a collection of L-asparaginase enzyme from different organisms and select a suitable enzyme based on its binding energy to L-asparagine.

The *E coli* L-asparaginase II has a functional form in a homotetramer having the molecular mass from 140 to 160 kDa. The monomers are 330 amino acid long and have two distinct domains. One is the larger N-terminal domain and the other is the smaller C-terminal domain. The two domains are connected by a 20-residue linker. The functional form of the enzyme is thought to contain five active sites [20].

Homology modeling is a technique used to generate a model from an amino acid sequence based on a template of a 3D structure of a closely related protein obtained via experimental data. It uses comparative protein structure modeling where the template and the query sequences are aligned and the query’s structure is predicted. According to Eswar et al [21], it has the following four major steps: fold assignment, which identifies similarity between the target and at least one known template structure; alignment of the target sequence and the template or templates; building a model based on the alignment with the chosen template or templates; and predicting model errors. We have used MODELLER 9.22 to model L-asparaginase sequence from the organisms that were selected, using the *E coli* L-asparaginase II (PDB ID: 1nns) as a template for generating all of them.

*E coli* and *Erwinia* L-asparaginases, the two commercially available forms of the therapeutic enzymes, have deficiencies in the aforementioned parameters. Thus, they show unsatisfactory results and side effects. In this research, we hope to find a better L-asparaginase from a different host organism for the commercial production of this therapeutic enzyme. We hypothesize that a host whose L-asparaginase amino acid sequence is distinct from that of the currently used organisms can be assumed to have markedly different properties. We can screen such a family or genus of host organisms and hope to find L-asparaginase that displays kinetic and binding properties that decrease the chances of immunogenic and allergic reactions making it more favorable for therapeutic use. We have used a phylogenetic tree-based approach to find such host organisms. A phylogenetic tree is an important bioinformatics tool that allows us to analyze the sequences of proteins, DNA, and RNA to find the historical and evolutionary relationship between the sequences. The nodes of a tree can be given values as support values for its reliability. These are called bootstrap values that give the expectation of that particular node in the many alternate trees generated by reruns of the same sequence data set [22]. Many algorithms for tree construction exist. Here, we have used the maximum likelihood (ML) algorithm in the MEGA bioinformatics tool to construct, bootstrap, and analyze our tree. The tree was used to look for hosts with evolutionarily distant L-asparaginase sequences, which can be screened for desired properties using docking tools.

## Methods

### Phylogenetic Tree Construction

To construct a phylogenetic tree, we retrieved the L-asparaginase B (asnB) protein sequence of *Escherichia coli k12* strain from the Uniprot [23] (UniProtKB-P00805 ASPG2_ECOLI). Microorganisms that are capable of producing the asnB based on the previous literature [24-27] were searched by doing blastp in the National Center for Biotechnology Information (NCBI) database [28]. Basic Local Alignment Search Tool (BLAST) is a sequence analysis tool that searches a database for sequences that are similar to a query sequence. Blastp is a variation of standard blast that searches a database of nonredundant and nonpatented sequences based on a query sequence. Blastp can be used to search a database for organisms that produce sequences that are the same or similar to our query sequence, helping us in compiling a list of known asnB-producing organisms that can be used for construction of our phylogenetic tree. The protein sequence of *E coli k12* asnB was used as the query sequence for blastp on a nr database resulting in a list of organisms that produced proteins of a similar sequence. The organisms with percentage identity greater than or equal to 30% were selected. The genomes of two types of organisms were searched for the presence of asnB. The first group of organisms were already characterized for the production of asnB protein. The other group of organisms included bacteria and archaea from various phyla [29] that represented the entire tree of life. A total of 101 sequences were retrieved after searching for asnB sequence in organisms given by the literature. Organisms with more than one asnB sequences were also retrieved and labeled as genus species 1, 2, or 3. The phylogenetic tree was then constructed in Mega-X software (Pennsylvania State University) [30], in which the alignment was done by Muscle. The following criteria were used to run a tree: statistical method: ML; test of phylogeny: bootstrap method; substitution type: amino acid; model or method: WAG model; rates among sites: gamma distributed with invariant sites, number of discrete gamma categories: 5; gaps or missing data treatment: partial deletion; site coverage cutoff: 95%; ML heuristic method: nearest neighbor interchange; initial tree for ML: make initial tree automatically (Default-NJ/BioNJ); branch swap filter: None; and number of threads: 3 [31]. In our method, we have used a sequence based on genetic or evolutionary distance for the construction of our tree.

### Homology Modeling

The organisms that were distantly placed in the phylogenetic tree with respect to *E coli* and *Erwinia* were chosen, and organisms whose enzymes were characterized in the literature were also chosen. To carry out homology modeling, the MODELLER 9.22 was used. The selected organism’s asnB sequence was used as the query while *E coli k12* asnB (“1nns”) [32] with a resolution of 1.95 Å was used as the reference template. Discrete optimization protein energy (DOPE) is an atomic distance–based scoring function used to access the quality of models produced from homology modeling, derived from a sample of native protein structures in PDB. Statistically optimized atomic potentials (SOAP) is another scoring function based on data from native protein structures used in the assessment of homology modeling results. For each organism, the structure with the lowest DOPE or SOAP assessment score and with the highest GA341 assessment score was selected [33]. Each protein’s model was then checked for protein structure stereochemistry including Ramachandran plot and Psi/Phi angles using PROCHECK. Further verification was done using WHATCHECK and ProSA-web [34].

### Active Site Prediction

After the validation of the model, active sites for each protein were determined using PyMol (Schrödinger, Inc) software [35]. The models built were superimposed to the 1nns structure, and then by aligning both model and 1nns sequences, the active site with reference to the 1nns active site was predicted. The active site of 1nns for L-asparagine is T(12), S(58), Q(59), T(89), and D(90) [36].

### Molecular Docking Studies

Docking of ligands, L-asparagine (derived from the PubChem website) with enzymes L-asparaginases (distant proteins from E coli and Erwinia and enzymes with measured Km value) was performed by using AutoDock Vina [37] conjugated with PyRx software (Sarkis Dallakian) [38]. The AutoDock tool’s graphic interface was used for the preparation of all the proteins (enzymes). Proteins were prepared by removing water, adding polar hydrogen, merging nonpolar, and adding Kollman charge. In the case of ligand, L-asparagine was retrieved from PubChem (Compound CID: 6267; molecular formula: C4H8N2O3; molecular weight: 132.12 g/mol) [39]. Energy minimization was done by the Universal Force Field using Open Babel (Open Babel Development Team) software [40] conjugated with PyRx. The grid parameter file and docking parameter file were set, and the grid points for auto grid calculations were set as 25 × 25 × 25 Å, with the active site residues in the middle of the grid box. The algorithm used in the overall process was the Lamarckian genetic algorithm, which was used to calculate protein-fixed ligand-flexible calculations [41].

### Interacting Atoms With Active Sites

Distant organisms’ asnBs with the best binding energies were selected. The docked protein and ligand files were run on ligPlot+ (European Bioinformatics Institute) software [42] for viewing the interacting atoms between ligands and proteins.

### Relation Between Km, k_cat_, and Binding Energy

To evaluate if the binding energy could predict the relative efficacy of the enzymes, Km and k_cat_ values from the literature were tabulated alongside binding energy. A total of 10 Km and 5 k_cat_ values were obtained from the literature for asnBs of different species. The line fitted plot was drawn using minitab [43], plotting binding energy on the x-axis and Km on the y-axis.

### Pairwise Sequence Alignment

Pairwise sequence alignment and comparison of three predicted optimal asnB enzyme sequences was done against the *E coli* asnB enzyme sequence using blastp (protein-protein blast) on Blast+ [28]. Scoring parameters used were BLOSUM62 matrix, gap penalties of 11 for existence, and 1 for extension.

## Results

### Deductions From the Phylogenetic Tree

**Figure 1:**
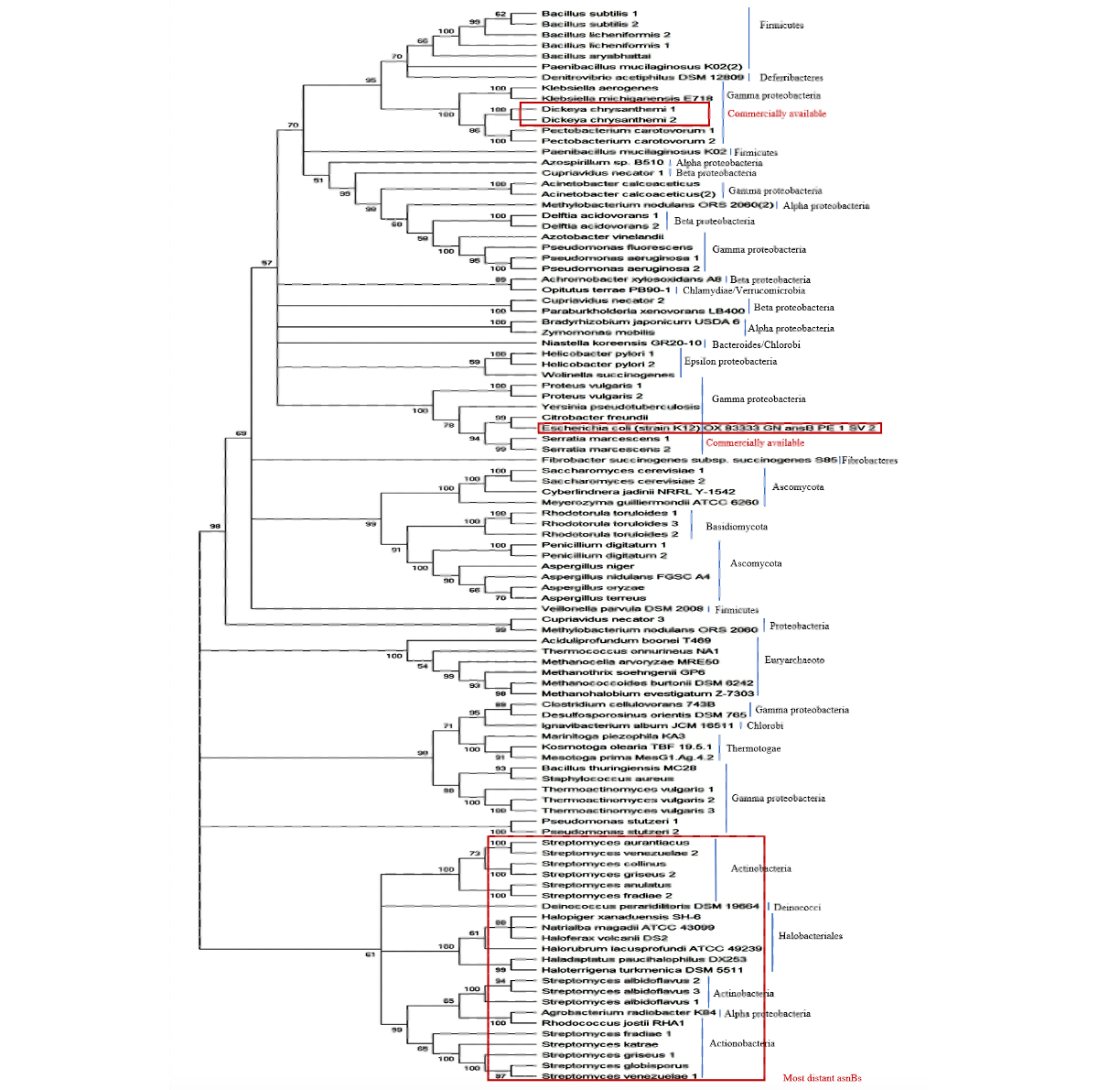
Phylogenetic tree of the total 101 sequences of asnBs using the maximum likelihood method. The top and middle portion of the tree under the red rectangle shows organisms that are currently used for the commercial production of asnBs for the treatment of acute lymphocytic leukemia. The bottom portion of the tree shows organisms that are most distant to E coli (mostly Actinobacteria), and their enzyme activity is yet to be discovered.

A list of asparaginase-producing organisms were compiled from the literature. Asparaginase II (asnB) homologs of these organisms were searched by protein blasting asnB from *E coli* against the nonredundant protein database of these organisms in NCBI. The organisms whose genomes are not sequenced were not used in this study. Additionally, the protein database of a wide variety of bacteria and archaea from different phylum were searched for the presence of asnB. The two lists were compiled to make up our list of a wide range of asnBs. A ML phylogenetic tree of 101 asnBs was drawn for these proteins using Mega X software using the parameters described in the Methods section. The resulting tree is shown in Figure 1. The phylum of bacteria, archaea, and fungi to which the proteins belong to is labeled on the right. Unlike most other proteins for which similar trees were drawn, there were minimal proteins from the same phylum that lay next to each other in the tree. When a similar tree was drawn for Ku protein in bacteria and beta clamp for bacteria, proteins from the same phylum tended to cluster together in the tree (unpublished data). Although some clustering is found for asnB tree, proteins from the same phylum are distributed throughout the tree, indicating extensive horizontal gene transfer. Among the list of asnBs that we have collected, the largest number of proteins comes from proteobacteria (alpha, beta, gamma, delta, and epsilon).

Besides predicting the origin and history of asparaginases, the tree is also useful in predicting which of the asnBs are closely related by evaluating which lie close together and which lie further apart. From the tree, the most common commercially used asnB from *E coli* lies somewhere in the center. The other commercially used asnB from *Erwinia* (nowadays called *Dickeya chrysanthami*) lies at the top of the tree. The asnBs that are most distant from these two commercially available asparaginases, and hence least likely to give an immunogenic reaction when these two give an immunogenic reaction, lie at the bottom of the tree. Of the 101 asnBs used in construction of the phylogenetic tree, 23 asnBs were selected as candidates for better enzyme activity due to them being the most evolutionarily distant from the commercially available asnBs. These have been labeled in Figure 1. Most of them lie in the *Streptomyces* genus and some are from archaea. Since most of the candidates in this group were *Streptomyces*, we decided to limit our list of potential asnB candidates to the 13 *Streptomyces* species in the list. Thus, we screened 13 potential species out of the 101 asnB-producing organisms we had found via blastp due to them being most evolutionarily distant from the organisms that produce commercially available asnBs.

Use of a phylogenetic tree is perfectly adequate for identifying organisms that produce asnBs that can be expected to have better activity and lower immunogenicity than commercially available asnBs. This is because there is a direct relationship between a protein’s sequence, structure, function, and immunogenicity. Therefore, asnBs that are evolutionarily distant to commercially available asnBs can be expected to have markedly different structure and can be expected to have potentially better activity than commercial variants. We can also expect evolutionarily distant asnBs to show different immunogenicity when compared to their commercial counterparts. The severity of immunogenic reaction from an antigen on an organism depends on the measure of its novelty. Immune response to a biological macromolecule is complex and dependent on many factors, a significant one being structure, which is dependent on sequence [44]. Two proteins that are evolutionarily different will also be structurally different and thus have different levels of immune responses. An example is that commonly used experimental antigen bovine serum albumin does not show immunogenic reaction when injected in cows but is actively immunogenic when injected into rabbits. Sidewise it would show enhanced reaction in chickens than in goats, for the reason that the latter is closely related to bovines. These analyses endorse that the greater the phylogenetic distances between two species, the greater the structural (and therefore the antigenic) divergence that can be found between them [45].

### Homology Modeling and Verification

**Figure 2:**
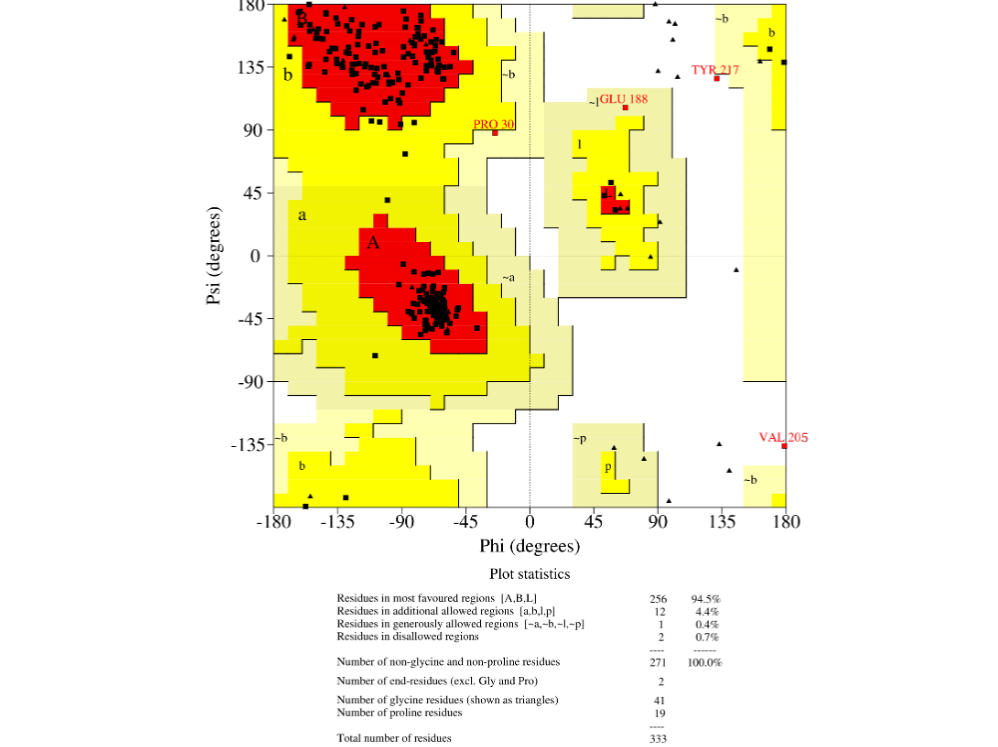
Ramachandran plot of *Streptomyces collinus*. The Ramachandran plot shows the phi-psi torsion angles for all residues (black cubes) in the structure (except those at the chain termini). Glycine residues are separately identified by triangles, as these are not restricted to the regions of the plot appropriate to the other sidechain types. The darkest red area indicates "core" regions representing the most favorable combinations of phi-psi values. The regions are labeled as follows: A (core alpha), L (core left-handed alpha), a (allowed alpha), l (allowed left-handed alpha), ~a (generous alpha), ~l (generous left-handed alpha), B (core beta), p (allowed epsilon), b (allowed beta), ~p (generous epsilon), and ~b (generous beta).

**Figure 3:**
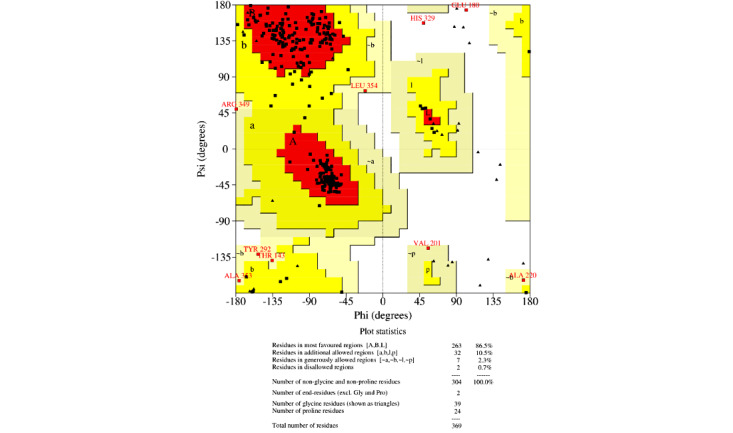
Ramachandran plot of *Streptomyces griseus* 1. The Ramachandran plot shows the phi-psi torsion angles for all residues (black cubes) in the structure (except those at the chain termini). Glycine residues are separately identified by triangles, as these are not restricted to the regions of the plot appropriate to the other side chain types. The darkest red area indicates the "core" regions representing the most favorable combinations of phi-psi values. The regions are labeled as follows: A (core alpha), L (core left-handed alpha), a (allowed alpha), l (allowed left-handed alpha), ~a (generous alpha), ~l (generous left-handed alpha), B (core beta), p (allowed epsilon), b (allowed beta), ~p (generous epsilon), and ~b (generous beta).

**Figure 4:**
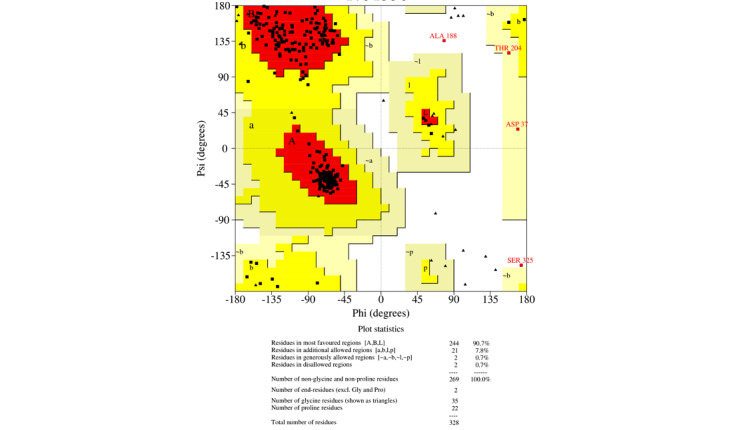
Ramachandran plot: *Streptomyces venezuelae* 2. The Ramachandran plot shows the phi-psi torsion angles for all residues (black cubes) in the structure (except those at the chain termini). Glycine residues are separately identified by triangles, as these are not restricted to the regions of the plot appropriate to the other sidechain types. The darkest red area indicates the "core" regions representing the most favorable combinations of phi-psi values. The regions are labeled as follows: A (core alpha), L (core left-handed alpha), a (allowed alpha), l (allowed left-handed alpha), ~a (generous alpha), ~l (generous left-handed alpha), B (core beta), p (allowed epsilon), b (allowed beta), ~p (generous epsilon), ~b (generous beta).

**Figure 5:**
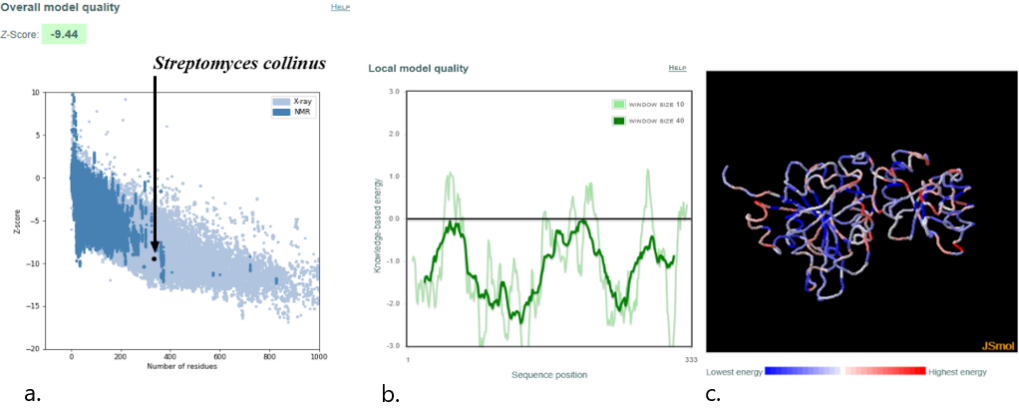
Validation of model *Streptomyces collinus*. (a) ProSA-web z scores of all protein chains in the Protein Data Bank determined by X-ray crystallography (light blue) or NMR (dark blue) with respect to their length. The black dot in the plot indicates that the model protein structure falls inside the range of the plot that contains the z score of all the experimentally determined proteins in the Protein Data Bank. The plot shows only chains with less than 1000 residues and a z score 10. The z scores of model proteins are highlighted as large dots. (b) Energy plot of model protein that indicates the local model quality by plotting energy as the function of the amino acid sequence. Generally, the portion in the positive region of the plot indicates the erroneous part of the structure. (c) Residues are colored from blue to red in the order of increasing residue energy. NMR: nuclear magnetic resonance.

**Figure 6:**
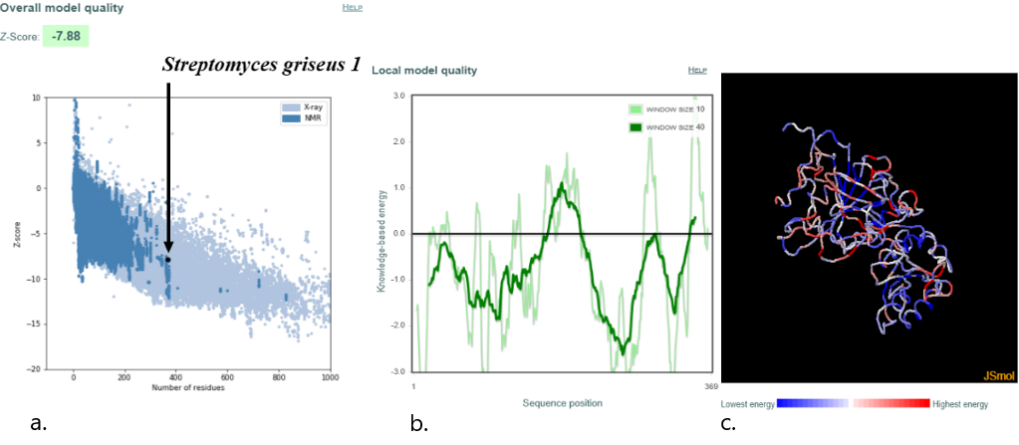
Validation of model: *Streptomyces griseus* 1. (a) ProSA-web z scores of all protein chains in the Protein Data Bank determined by X-ray crystallography (light blue) or NMR spectroscopy (dark blue) with respect to their length. The black dot in the plot indicates that the model protein structure falls inside the range of the plot that contains the z score of all the experimentally determined proteins in the Protein Data Bank. The plot shows only chains with less than 1000 residues and a z score of 10. The z scores of model proteins are highlighted as large dots. (b) Energy plot of model protein that indicates the local model quality by plotting energy as the function of the amino acid sequence. Generally, the portion in the positive region of the plot indicates the erroneous part of the structure. (c) Residues are colored from blue to red in the order of increasing residue energy. NMR: nuclear magnetic resonance.

**Figure 7:**
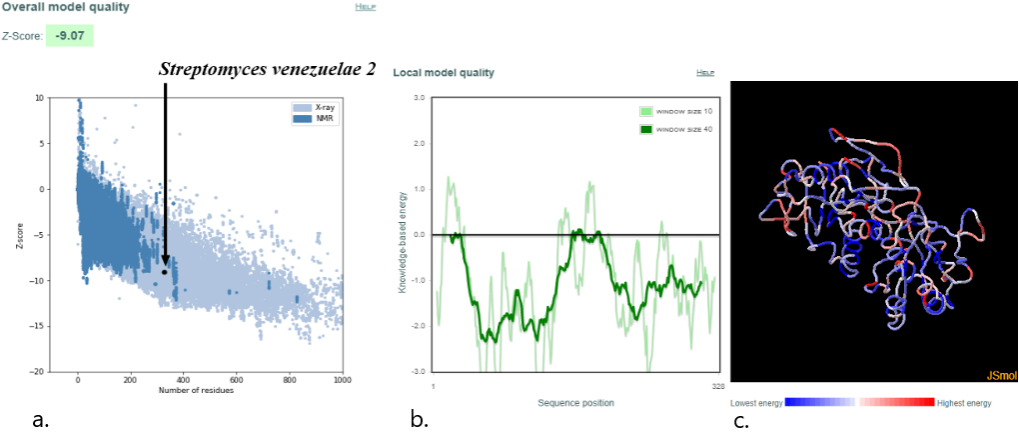
Validation of model: *Streptomyces venezuelae* 2. (a) ProSA-web z scores of all protein chains in the Protein Data Bank determined by X-ray crystallography (light blue) or NMR spectroscopy (dark blue) with respect to their length. The black dot in the plot indicates that the model protein structure falls inside the range of the plot that contains the z score of all the experimentally determined proteins in the Protein Data Bank. The plot shows only chains with less than 1000 residues and a z score 10. The z scores of model proteins are highlighted as large dots. (b) Energy plot of model protein that indicates the local model quality by plotting energy as the function of the amino acid sequence. Generally, the portion in the positive region of the plot indicates the erroneous part of the structure. (c) Residues are colored from blue to red in the order of increasing residue energy.

For homology modeling, MODELLER 9.22 (University of California, San Francisco) software was used, and five models were built for each protein, among which the model with the lowest DOPE was selected. This software uses an inbuilt DOPE function to access the quality of all the models that were made. The model that was selected according to the lowest DOPE scores was validated using Ramachandran plot. A Ramachandran plot of the three best organisms that lie distant to the *E coli* and have a better binding affinity toward L-asparagine than *E coli* and *Dickeya chrysanthami* are shown in Figures 2-4. The plot shows 94.5% (256/271) of residues in most favored regions, 4.4% (12/271) in additional allowed regions, 0.4% (1/271) residues in generously allowed regions, and 0.7% (2/271) residues in disallowed regions for *Streptomyces collinus* (Figure 2); 86% (263/304) of residues in most favored regions, 10.5% (32/304) in additional allowed regions, 2.3% (7/304) residues in generously allowed regions, 0.7% (2/304) residues in disallowed regions for *Streptomyces griseus* 1 (Figure 3); and 90.7% (244/269) of residues in most favored regions, 7.8% (21/269) in additional allowed regions, 0.7% (2/269) residues in generously allowed regions, and 0.7% (2/269) residues in disallowed regions for *Streptomyces venezuelae 2* (Figure 4). More than 99% of residues in the allowed region given by the Ramachandran plot indicate a very good model. Furthermore, the Ramachandran *z* scores calculated by WHATCHECK (–0.245, –1.024, and –0.830 for *S collinus*, *S griseus* 1, *S venezuelae* 2, respectively) fall on the accepted region [46] and were allowed by the WHATCHECK. The structures were finally validated using ProSA-web server. This server gives the *z* score, which indicates the overall model quality and measures the deviation of the total energy of the structure with respect to an energy distribution derived from random conformations [47]. The *z* scores given by the server (–9.44, –7.88, and –9.07 for *S collinus, S griseus 1,* and *S venezuelae 2*, respectively) fall inside the range of the plot (black dot) that contains the *z* scores of all the experimentally determined protein in the PDB (X-ray, nuclear magnetic resonance; part a of Figures 5-7). The energy plot (part b of Figures 5-7) indicates the local model quality by plotting energy as the function of the amino acid sequence. Generally, the portion in the positive region of the plot indicates the erroneous part of the structure. We can conclude from the plot that the structure is feasible or accepted as overall residue energies fall under the negative part of the plot. The colored 3D structure of the proteins (part c of Figures 5-7) shows that the portion in red color is of high energy and the portions with the blue color are of low energy [34]. Validation of all other structures used in the experiment is in Multimedia Appendix 1. Most of the active site residues are conserved in every model made by MODELLER 9.22 in reference to the 1nns structure, which also signifies that good models were made during the process and can proceed toward the docking (Table 1).

**Table 1:** Predicted active sites of proteins of organisms that were distant to the *E coli* and organisms whose Km has been determined experimentally (described elsewhere in the paper).^a^

Organisms	Predicted active site residues
*Escherichia coli*	T(34), S(80), Q(81), T(111), D(112)
*Streptomyces globisporus*	I(12), S(61), S(62), T(94), D(95)
*Streptomyces venezuelae* 1	I(12), S(61), S(62), T(94), D(95)
*Streptomyces griseus* 1	T(20), S(61), S(62), T(94), D(95)
*Streptomyces katrae*	T(12), S(53), P(54), T(86), D(87)
*Streptomyces fradiae*	A(12), G(43), A(44), T(75), D(76)
*Streptomyces albidoflavus* 1	T(12), M(62), R(63), T(94), D(95)
*Streptomyces albidoflavus* 2	T(12), M(62), R(63), T(94), D(95)
*Streptomyces albidoflavus* 3	T(12), R(63), L(64), T(94), D(95)
*Streptomyces fradiae* 2	T(8), S(50), Y(51), T(83), D(84)
*Streptomyces collinus*	T(16), S(63), L(64), T(94), D(95)
*Streptomyces griseus* 2	T(16), P(60), G(61), T(94), D(95)
*Streptomyces aurontiacus*	T(13), S(54), L(55), T(83), D(84)
*Streptomyces venezuelae* 2	T(12), —, —, T(79), D(80)
*Pectobacterium carotovorum* 1	T(34), S(81), E(82), T(114), D(115)
*Dickeya chrysanthami (Erwinia)* 1	T(36), S(83), E(84), T(116), D(117)
*Bacilus aryabhattai*	T(55), S(102), Q(103), S(135), D(136)
*Bacillus Licheniformis* 1	T(62), S(109), Q(110), T(142), D(143)
*Bacillus subtilis* 1	T(61), S(108), T(109), T(141), D(142)
*Delftia acidovorans* 1	T(62), S(109), E(110), T(142), D(143)
*Azotobacter vinelandii*	T(45), S(92), E(93), T(125), D(126)
*Dickeya chrysanthami (Erwinia)* 2	T(36), S(83), E(84), T(116), D(117)
*Helicobacter pylori* 1	T(34), S(80), Q(81), T(113), D(114)
*Pseudomonas stutzeri* 1	—, S(80), D(81), T(113), D(114)
*Pseudomonas stutzeri* 2	—, S(80), D(81), T(113), D(114)
*Bacillus subtilis* 2	T(61), S(108), T(109), T(141), D(142)
*Bacillus licheniformis* 2	T(63), S(110), T(111), T(143), D(144)
*Delftia acidovorans* 2	T(62), S(109), E(110), T(142), D(143)
*Helicobacter pylori* 2	T(34), S(80), Q(81), T(113), D(114)
*Pectobacterium carotovorum* 2	T(34), S(81), E(82), T(114), D(115)

^a^Five amino acids were conserved, which has been termed a pentad in this paper. The letter represents the amino acid involved in the active site, the number in parenthesis represents the position of the amino acid. When no amino acid homology was found, the site was left blank with an em dash.

### Active Site of asnBs

Along with the 1nns structure of *E coli* asnB, obtained from pdb, comes the description of active site amino acid residues. Using aspartate as a surrogate for asparagine, the active sites have been predicted. For the full-length protein, the active site contains 5 amino acid residues: T(34), S(80), Q(81), T(111), and D(112). These 5 residues can be called a pentad. A table with these pentad residues has been constructed for asnBs of other organisms (Table 1). Four of the five residues—T(34), S(80), T(111), and D(112)—are highly conserved across species (Table 1).

### Km, k_cat_, and Binding Energies of asnBs

To further predict which list of asnBs would be most useful to treat acute lymphoblastic leukemia, binding energies were calculated using docking software. First, using a 1nns structure of *E coli* asnB, structures of unsolved asnBs were predicted using homology modeling These structures were docked to asparagine to calculate binding energy. To evaluate if the binding energy could predict the relative efficacy of the enzymes, Km and k_cat_ values from the literature were tabulated alongside binding energy (Table 2). A total of 10 Km values were obtained from the literature for asnBs of different species. For the species with only 1 Km value—*Escherichia coli*, *Azobacter vinelandi*, and *Bacillus aryabhattai*—comparison between the relationship of Km and binding energy was easy. When Km value increased, binding energy decreased. Species with the highest binding energy, *E coli*, also had the lowest Km value. Species with the lowest binding energy, *Bacillus aryabhattai*, had the highest Km value.

**Figure 8:**
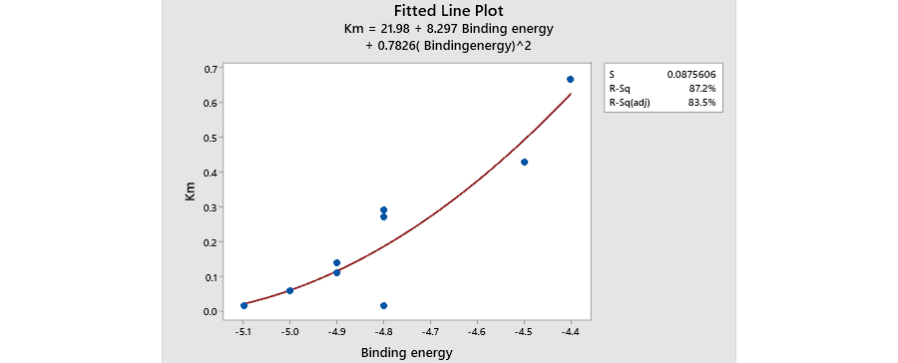
Relation between Km and binding energy of enzyme toward L-asparagine. The fitted line plot shows that Km and binding energy are inversely proportional to each other. The more negative the binding energy, the less the Km value is. More negative binding energy and less Km signifies the greater affinity of an enzyme toward the substrate. All the enzymes' Km and Binding energy shows how they are inversely proportional to each other except one, which is the enzyme from Bacillus licheniformis 1 (0.014mM Km at –4.8 kcal/mol). We were also unable to confirm that the sequence of the enzyme that was used to calculate the Km value [48] and the sequence of the enzyme used in this experiment was the same.

However, six species contained two asparaginases. From the literature, specific Km values could be assigned to specific asnBs (ie, sequence of protein used to calculate the Km experimentally and sequence of protein used to calculate the binding energy were the same). Those asnBs are marked in the table. *Dickeya chrysanthami* 2, *Heliobacter pylori* 1, and *Bacillus subtilis* 1 had known Km values that were assigned next to them on the table. Similarly, using docking, separate binding energies could be calculated for each asnB protein. In species where two asnBs are available, the Km value measured for the species is assigned to asnB that most closely forms an inverse relationship with the binding energy. For example, *Pseudomonas stutzeri* has two asnBs with binding energies of –5.1 Kcal/mol and –4.9 kcal/mol. Since its Km value is high, the asnB with low binding energy was assigned this Km, although this could not be verified experimentally. When all values were assigned, a clear inverse relationship between Km and binding energy emerged. The binding energies of asnB to asparagine ranged from –5.1 kcal/mol to –4.4 kcal/mol, which are relatively high values of binding in AutoDock Vina software. No relationship could be discerned for k_cat_ value and binding energy. To be able to compare Km value to binding energies, plots were drawn. A smooth curve was fitted (Figure 8).

**Table 2:** Km value, k_cat_ value (retrieved from the literature), and binding energy (calculated by AutoDock Vina) of the enzyme, asnB, toward L-asparagine.

Organism	Michaelis constant value from literature (mM)	Measured k_cat_ values from literature (s^–1^)^a^	Binding affinity calculated from docking (kcal/mol)	References
*Bacillus licheniformis* 1	0.014	2.68 × 10^3^	–4.8	[48]
*Escherichia coli^b^*	0.015	2.4 × 10^1^	–5.1	[49]
*Deftia acidovorous^b^*	0.015	—^c^	–5.1	[50]
*Dickeya chrysanthami* 2^b^	0.058	23.8 × 10^3^	–5.0	[51]
*Azobacter vinelandi^b^*	0.11	—	–4.9	[52]
*Pseudomonas stutzeri* 2	0.14	—	–4.9	[53]
*Bacillus aryabhattai^b^*	0.257	—	–4.8	[54]
*Helicobacter pylori* 1^b^	0.29	19.26 +/– 0.56	–4.8	[55]
*Bacillus subitilis* 1^b^	0.43	—	–4.5	[56]
*Pectobacterium carotovorum* 1	0.657	2.751 × 10^3^	–4.4	[57]
*Dickeya chrysanthami* 1	—	—	–4.4	—
*Bacillus licheniformis* 2	—	—	–4.6	—
*Pseudomonas stutzeri* 1	—	—	–5.1	—
*Deftia acidovorous* 2	—	—	–5.0	—
*Bacillus subtilis* 2	—	—	–5.0	—
*Pectobacterium carotovorum* 2	—	—	–4.7	—
*Heliobacter pylori* 2	—	—	–5.1	—

^a^k_cat_ values demonstrate no relationship to the binding energy.

^b^For 6 species, corresponding Km values and binding energies are known (ie, the sequence of protein used to calculate the Km experimentally and the sequence of protein used to calculate the binding energy were the same). For four other species, the Km value that best fit the binding energy value was randomly assigned. The six Km values are perfectly inversely correlated to binding energies.

^c^Experimental data is not available for these particular organisms in the literature.

### Finding an Optimal asnB

**Figure 9:**
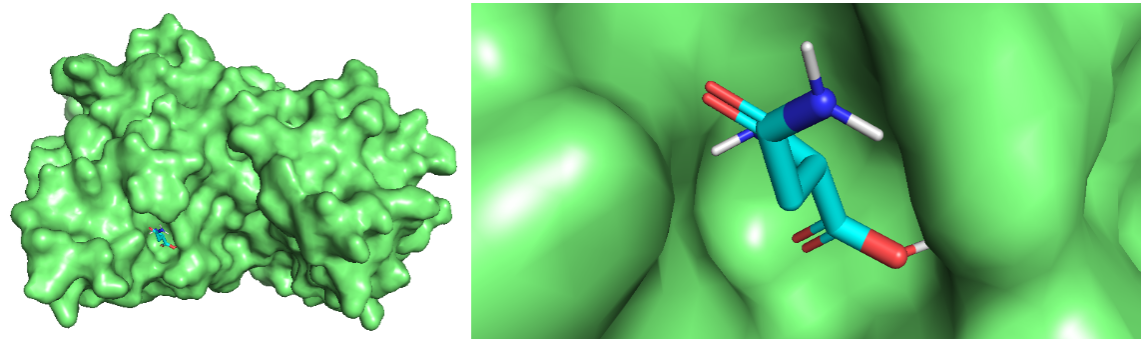
Docked structure of *Escherichia coli* asnB and *L-asparagine*. L-asparagine is seen to be completely impended in the catalytic pocket of the enzymes.

**Figure 10:**
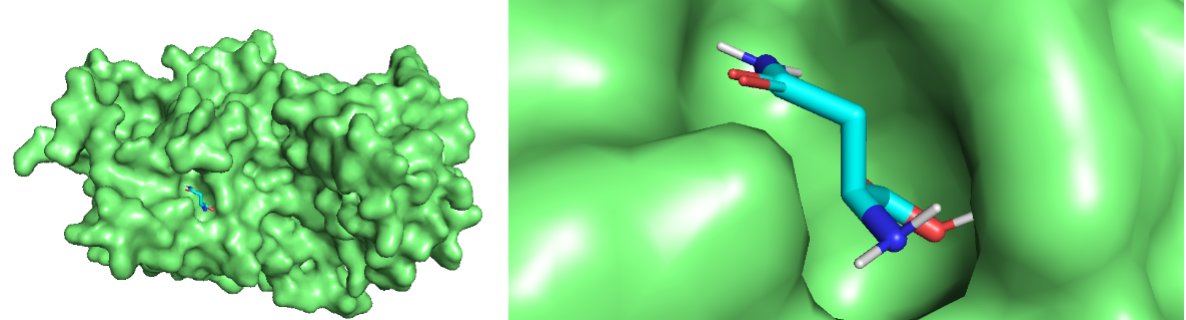
Docked structure of *Streptomyces griseus* 1 asnB and *L-asparagine*. L-asparagine is seen to be completely impended in the catalytic pocket of the enzymes.

**Figure 11:**
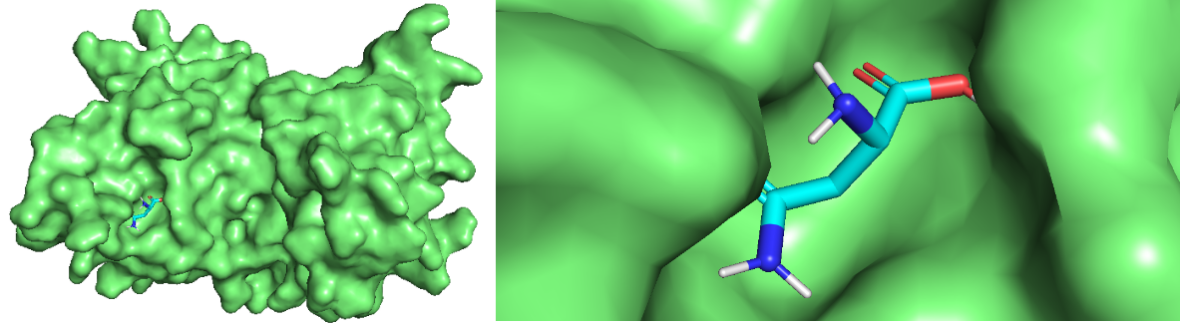
Docked structure of *Streptomyces venezuelae* 1 asnB and *L-asparagine*. L-asparagine is seen to be completely impended in the catalytic pocket of the enzymes.

**Figure 12:**
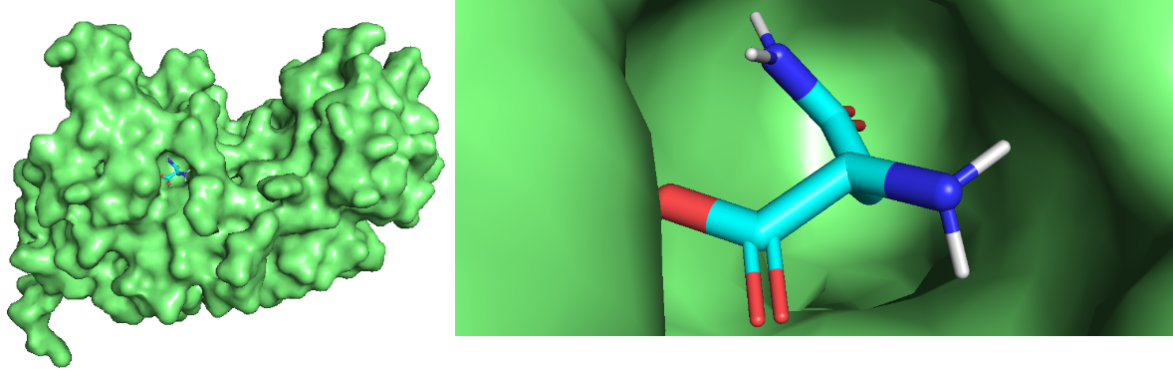
Docked structure of *Streptomyces collinus* asnB and *L-asparagine*. *L-asparagine* is seen to be completely impended in the catalytic pocket of the enzymes.

For 13 asnBs that are most distant from *E coli* and *Erwinia* asparaginase, binding energies were calculated using docking (Table 3). The proteins for which binding energy were calculated are *Streptomyces albidoflavus* 1, 2, and 3; *Streptomyces aurantiacus*; *Stereptomyces collinus*; *Streptomyces fradiae* 1 and 2; *Streptomyces globisporus*; *Streptomyces griseus* 1 and 2; *Streptomcyces katrae*; and *Streptomyces venezuelae* 1 and 2. Out of these 13 proteins, 3 asnBs—*Stereptomyces collinus*, *Streptomyces griseus* 1, and *Streptomyces venezualae* 2—showed biding energy of –5.3 kcal/mol, –5.3 kcal/mol, and 5.2 kcal/mol, respectively, higher than *E coli* anB. Docked structures are shown in Figures 9-12. These asparaginases can be further cloned and tested for Km and k_cat_ values.

**Table 3:** Binding energy of distant organism’s asnB and L-asparagine.

Organisms	Binding affinity calculated from docking (kcal/mol)
*Streptomyces albidoflavus* 1	–4.8
*Streptomyces albidoflavus* 2	–4.8
*Streptomyces albidoflavus* 3	–4.5
*Streptomyces aurantiacus*	–4.2
*Streptomyces collinus* ^a^	–5.3
*Streptomyces fradiae* 1	–4.9
*Streptomyces fradiae* 2	–4.9
*Streptomyces globisporus*	–4.2
*Streptomyces griseus* 1^a^	–5.3
*Streptomyces griseus* 2	–4.6
*Streptomyces katrae*	–4.9
*Streptomyces venezuelae* 1	–4.8
*Streptomyces venezuelae* 2^a^	–5.2

^a^*Streptomyces collinus*, *Streptomyces griseus* 1, and *Streptomyces venezuelae* 2 asnBs have –5.3 kcal/mol, 5.3 kcal/mol, and 5.2 kcal/mol binding energy, respectively, which is greater than the *E coli* and *Dickeya chrysanthami* –5.1 and –5.0 kcal/mol, respectively, which indicate that these organisms’ asnB have a greater affinity toward the L-asparagine.

### Pairwise Sequence Alignment

**Figure 13:**
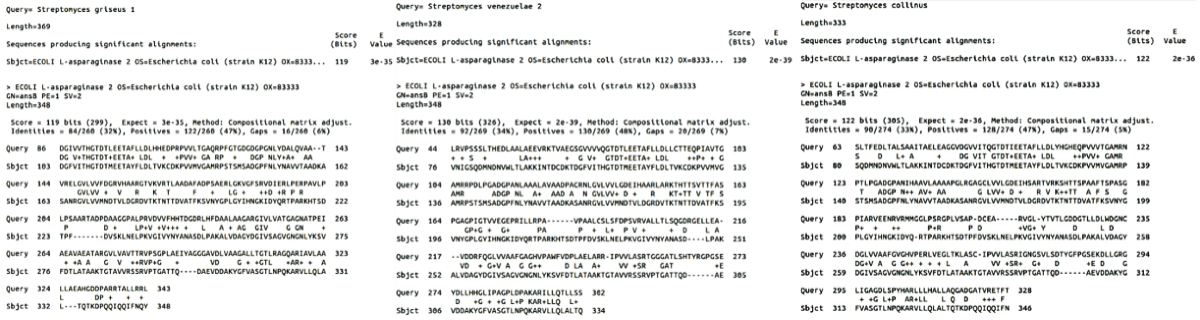
Sequence alignment results for Streptomyces collinus, Streptomyces griseus 1, and Streptomyces venezuelae 2 asnB sequences with the E coli asnB sequence. The query sequence is displayed above the subject. Starting and ending amino acid positions for each row are given for both query and subject. The score, E values, the percentage of positive hits, and the percentage of gaps are given above the alignment diagram.

We also compared the amino acid sequence of the three optimal asnBs selected with that of *E coli* asnB sequence. *Streptomyces venezuelae* 2 showed the highest alignment score of 130 with 34% sequence identity to *E coli* asnB. *Streptomyces collinus* showed 33% identity with *E coli* and an alignment score of 122. *Streptomyces griseus* 1 had the lowest alignment score of 119 and sequence identity of 32% among the three optimal asnBs selected. Conversely, *Streptomyces griseus* 1 had the lowest E value (3 × 10^–35^) compared to *Streptomyces venezuelae* 2 (2 × 10^–39^) and *Streptomyces collinus* (2 × 10^–36^). All of them had a similar percentage of gaps when aligned with the query sequence shown in Figure 13.

### Interaction With Active Sites

**Figure 14:**
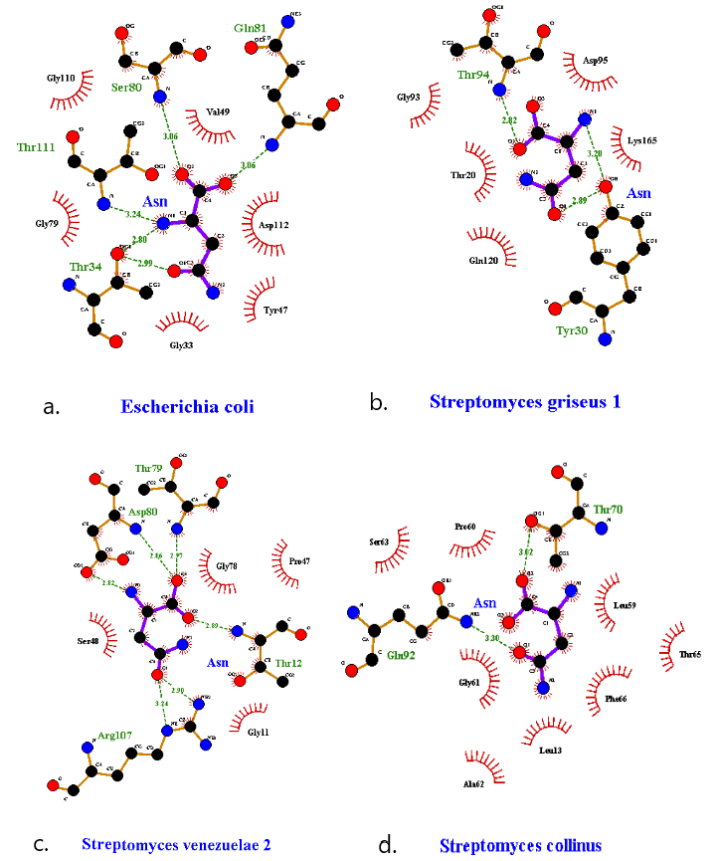
LigPlot of interacting atoms of *E coli* and selected three organisms. (a) *Escherichia coli*, (b) *Streptomyces griseus* 1, (c) *Streptomyces venezuelae* 1, (d) *Streptomyces collinus* enzymes, and *L-asparagine* (Asn).

A LigPlot showing active site interactions of asnB and asparagine was constructed and is shown in Figure 14. The active site of *E coli* asnB contains all 5 active site residues. Four of those residues—T(34), S(80), Q(81), and T(111)—form direct hydrogen bonding with asparagine. D(112), unlike in the 1nns active site predicted by pdb, does not form a hydrogen bond and only stays in the active site as a hydrophobic interactor in our LigPlot model. As 1nns is the structure complexed with aspartic acid (D), a closer inspection of the active site interactions in the 1nns predicted in the pdb website and our LigPlot model show some similarities and some variations.

LigPlot showing active site interactions of asnB and asparagine was constructed and shown in Figure 14. In *Streptomyces griseus* 1 asnB, 3 amino acid residues—T(20), T(94), and D(95)—of the pentad (out of five predicted residues) interacts with asparagine (Figure 14). Out of three residues, only one residue T(94) is involved in the formation of a hydrogen bond, whereas two other residues form a hydrophobic interaction with asparagine. Y(30) forms another hydrogen bond with asparagine. Only 3 of the pentads were detected in *Streptomyces venezuelae* 2. All three amino acids form an H-bond with asparagine. Additionally, R(107) forms a hydrogen bond with asparagine (Figure 14).

As for *Streptomyces collinus* asnB, 4 of the catalytic pentad residues—T(16), L(64), T(94), and D(95)—are absent at the catalytic site interaction with asparagine. Only S(63) is present in the active site. When the ligand was docked to the *Streptomyces collinus* asnB predicted active site with the grid box size 25 × 25 × 25 Å, AutoDock software automatically detected that there was another catalytical pocket present adjacent to the predicted one with almost the same interacting residues (Figure 14) as predicted but with the different position that gives the binding energy of –5.3 kcal/mol, where T(70) and Q(92) contributes on hydrogen bonding and other residues are involved in hydrophobic interaction. This binding site is shown in Figure 14 and is visibly almost the same but in a different position from all predicted active site residues.

## Discussion

Rapid and cost-effective screening of enzymes is a common undertaking in enzymology. Industrially produced enzymes have a role in a wide range of functions in pharmaceutical, food, biofuel, and chemical industries. Such enzymes are often screened from novel organisms in the soil, water, or other resources. Many of the commercially useful enzymes have been discovered through such screens. The fungus that produces cellulase, *Trichoderma reesei*, was isolated from garments and canvas that was degraded in the Solomon Islands during the Second World War [58]. Similarly, most of the alpha amylases used in the industry find their source in Bacillus [59]. Asparaginase that is used as an anticancer agent is derived from *E coli* and *Erwinia*. Most of these microorganisms have been discovered from simple screens developed for certain enzymes. This does not necessarily mean that these enzymes have the most optimal sequences for activity. This is because the screen could have easily missed out on better sequences that are not as well expressed in native cells. If these better sequences could be discovered, they would be easily cloned into amenable expression systems, expressed in high numbers, and used for industrial purposes.

In this paper, we have developed a method to in silico screen for the sequence with the best enzymatic activity. Since asnB is one of the most widely screened and studied enzymes, we chose to in silico predict the optimal sequence for its production. The first task was to collect a list of sequences from which optimal sequences could be predicted. This task has been made easier in recent years by an explosion in the number of genomes of organisms sequenced. It has become easy to discover homologous proteins in different phyla and in different domains of life. We collected a total of 101 sequence homologs of asnB from different phyla in bacteria, archaea, and eukarya. Using these 101 sequences, an ML phylogenetic tree was constructed. The tree served two purposes. First, it helped us predict the evolution and history of the asnB protein. Since proteins from the same phylum tend to congregate little in the tree, it can be predicted that there was a lot of horizontal gene transfer during the evolution of asnB. Less than half the species we searched had asnB sequences, indicating the lack of the enzyme’s universal presence in different organisms. Second, the tree helped pick sequences that were most distant and hence least likely to cause immunogenicity when both *E coli* and *Erwinia* asnBs showed immunogenicity. *E coli*, being one of the most studied model organisms, was the obvious first choice as a source of asnB. There is no clear indication in the literature as to why *Erwinia* was chosen as the second source of asnB, but the tree we have drawn confirms that *Erwinia* as a source was a wise choice since *Erwinia* asnB lies at one end of the tree distant to *E coli* asnB that lies around the center of the tree. The organisms we have zeroed in on are distant compared to *Erwinia* and *E coli*, and mostly lie in the *Streptomyces* genus.

As we can see, phylogenetic analysis can provide valuable insight about our protein of interest. Phylogenetic methods have been previously used successfully for studying L-asparaginase given its importance in the therapeutic setting. These methods have proven useful in identifying similarities between asnBs from different organisms based on the evolutionary relationship of their sequences, allowing researchers to group together organisms producing asnBs at a molecular level. This has led to discoveries regarding important amino acids and sequences of the L-asparaginase enzyme [60]. Information gleamed from phylogenetic analysis is not only useful in understanding the genetic variation and history of a protein across various organisms but also for identifying organisms that may produce more optimal proteins than those that are currently used, especially for commercially important proteins. Researchers have used them to identify clades with specific amino acid sequences that are also found in *E coli*. This information was then used to short list candidates for in silico screening for alternative L-asparaginase using docking [61].

Molecular modeling and docking have proven adequate for studies involving screening for alternative L-asparaginase candidates and optimization of this enzyme. They have been successfully used in previous studies for identifying alternative organisms for higher production of L-asparaginase candidates. These studies have also been validated using *in vitro* experimental work on the identified candidates [62]. Similarly, docking has been used in screening for L-asparaginase enzymes that have better activity toward asparagine and reduce its glutaminase side activity as well [63]. We used homology modeling and virtual docking in our method to identify enzymes with better binding energy than the commercially available asnBs produced from *E coli* and *Erwinia*. The candidates we zeroed in on using the phylogenetic tree were modeled using homology modeling and their binding energy to our substrate, asparagine, calculated using docking. Of the 13 potential candidates we had identified from the tree, 3 of them, *Streptomyces griseus* 1, *Streptomyces venezuelae* 2, and *Streptomyces collinus*, were deemed to be better than the commercially available option.

Additionally, we wanted to develop an in silico tool to predict the reaction kinetics of individual enzymes. To that end, we relied on molecular modeling and docking approaches. Although reaction kinetics is defined by different parameters like Km, k_cat_, maximum velocity (Vmax), and specificity constant (k_cat_/Km), Km is often the most widely measured quantity. This turned out to be the case for asnBs as well. From the literature, 10 Km values corresponding to asnBs from different species were discovered, while only 4 k_cat_ values were discovered. We set out to discover if the sequence of asnB can predict Km value without having to determine it experimentally. Through homology modeling, we predicted the structures of asnBs with known Km. After that, asparagine (the substrate) was docked onto the predicted asnB structures, and the binding energy was calculated. This binding energy was compared to the measured Km values to detect a correlation. Out of 10 species for which Km is known, only in 6 species (*Escherichia coli*, *Deftia acidovorous*, *Dickeya chrysanthami* 2, *Azobacter vinelandi*, *Pseudomonas stutzeri*, *Bacillus aryabhattai*, *Helicobacter pylori* 1, and *Bacillus subitilis* 1) could Km be definitely assigned to a certain sequence. A clear inverse relationship between Km value and binding energy emerged. A higher Km value corresponded to lower binding energy.

This finding makes sense according to a definition of Km. The Michaelis-Menten kinetics is derived using the following equation:



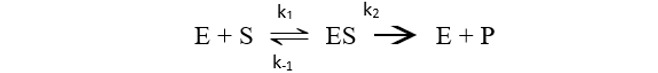



Where E is the enzyme, S is the substrate, ES is the enzyme-substrate complex, P is the product, k_1_ is the rate of forward reaction during the formation of ES complex, k_–1_ is the rate of backward reaction during ES dissociation into E and S, and k_2_ is the rate of reaction for the dissociation of ES complex into E and P. From this equation, Km is defined as (k_2_ + k_–1_) / k_1_. When k_2_ << k_–1_ under the rapid equilibrium assumption, K_m_ = k_–1_ / k_1_. Thus, Km is equal to the dissociation constant. There is also a relationship between the dissociation constant and binding energy—deltaG (binding energy) is proportional to –lnKm. However, when lnKm is plotted against binding energy, a linear fit graph was not obtained (data not shown). However, the negative relationship between Km and binding energy makes sense from this equation [64].

This result demonstrates that if binding energies can be compared among homologs, the homolog with the highest binding energy will give the lowest Km value. This can be used to predict the enzyme sequence that will give the lowest Km value. In this paper, the binding energies of asnBs from various Streptomyces species were calculated to obtain the one with the highest binding energy. Of the 13 asnBs, 3 give biding energy of –5.3 kcal/mol and –5.2 kcal/mol with asparagine. asnBs from *Streptomyces griseus*, *Streptomyces collinus*, and *Streptomyces venezuelae* gave these values. These values are higher than the binding energy of *E coli* and *Erwinia* asnBs. We can expect the kinetics of the enzyme produced from Streptomyces species to be better than those of commercially available asparaginase, making it a valuable target for cloning.

For the three optimal asnBs and *E coli* asnB, a LigPlot diagram of the active site along with interacting aspargine was drawn. It was demonstrated in *E coli* that the catalytic pentad residues were actively involved in bonding. Four of the five active-site residues formed hydrogen bonds, whereas one stayed in the active site forming hydrophobic interaction. Although the residues interacting are the same in the active site published by pdb site, different amino acid residues form hydrogen bonds with asparagine at different locations from the one given in the LigPlot in this paper. This is in line with the idea that the exact mechanism of asparaginase catalysis is not figured out, though it is predicted that the mechanism for type I and type II asparaginases will be conserved [65]. Two different mechanisms have been proposed for asparaginase catalysis. One mechanism describes double displacement, where the ammonia in asparagine is first displaced by the enzyme before the enzyme attached to asparagine is again displaced by water. The second mechanism describes the single displacement where water directly displaces ammonia from asparagine. There are contrary experimental and theoretical predictions for the validity of the two models [65,66].



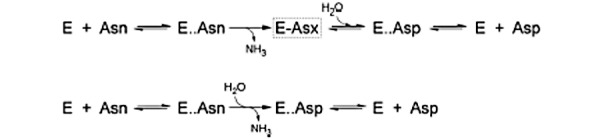



From the LigPlot of *Streptomyces griseus* 1 and *Streptomyces venezuelae* 2, it can be demonstrated that three of the pentad residues are present in the active site. This shows that the active site in these distant species is conserved. It has been predicted that one of the two threonines acts as a nucleophile in the double displacement mechanism. Conservation of both threonines suggests that this could indeed be the case. A dynamic simulation modeling rather than the static docking modeling we have carried out might give a clearer answer to the active sites involved, the catalytic mechanism, and the relevant nucleophiles and electrophiles.

Thus, we have devised an in silico method to predict the enzyme kinetics (Km value) from a sequence of an enzyme along with being able to screen for optimal alternative asnBs against acute lymphoblastic leukemia. Our method uses sequence-based phylogenetic analysis to zero in on a small number of candidates on which virtual docking can be used to identify a set of optimal enzymes that may be better than those that are commercially used. In this paper, we have shown the effectiveness of our method for identifying enzymes that are more optimal than a known commercial variant. We have also validated the effectiveness of this method to predict Km values of asparaginase II with a high degree of accuracy. This method is applicable not only to asparaginases but also to a slew of other industrial proteins such as amylases, cellulases, and many others. In the future, it will be worthwhile to apply this technique to the prediction of Km and the selection of industrially valuable sequences of other enzymes. We have predicted three possible highly promising L-asparaginase II enzymes produced by three Streptomyces species. The next step will be to verify using cloning if these sequences give a low Km value.

## Appendix

Multimedia Appendix 1 Supplementary file.

## Abbreviations

BLAST: Basic Local Alignment Search Tool
DOPE: discrete optimization protein energy
ML: maximum likelihood
NCBI: National Center for Biotechnology Information
SOAP: statistically optimized atomic potentials
